# Low serum uromodulin levels and their association with lupus flares

**DOI:** 10.1371/journal.pone.0276481

**Published:** 2022-10-27

**Authors:** Bonilla-Lara David, Gamez-Nava Jorge Ivan, Perez-Guerrero Edsaul Emilio, Murillo-Saich Jessica Daniela, Contreras-Haro Betsabe, Vazquez-Villegas Maria Luisa, Fajardo-Robledo Nicte Selene, Aguilar-Chavez Erika Anita Guadalupe, Saldaña-Cruz Ana Miriam, Celis Alfredo, Nava-Valdivia Cesar Arturo, Hernandez-Corona Diana Mercedes, Cardona-Muñoz Ernesto German, Laura Gonzalez-Lopez

**Affiliations:** 1 Programa de Doctorado en Farmacología, Instituto de Terapéutica Experimental y Clínica, Departamento de Fisiología, Centro Universitario de Ciencias de la Salud, Universidad de Guadalajara, Guadalajara, Jalisco, México; 2 Programa de Doctorado en Salud Publica, Departamento de Salud Pública, Centro Universitario de Ciencias de la Salud, Departamento de Salud Pública, Universidad de Guadalajara, Guadalajara, Jalisco, México; 3 Research Group of Factors Related to Therapeutic Outcomes in Autoimmune Diseases, Instituto de Terapéutica Experimental y Clínica, Departamento de Fisiología, Centro Universitario de Ciencias de la Salud, Universidad de Guadalajara, Guadalajara, Jalisco, México; 4 Instituto de Investigación en Ciencias Biomédicas, Centro Universitario de Ciencias de la Salud, Universidad de Guadalajara, Guadalajara, México; 5 Department of Medicine, School of Medicine, University of California, San Diego, California, United States of America; 6 División de Ciencias de la Salud, Centro Universitario de Tonalá, Universidad de Guadalajara, Tonalá, Jalisco, México; 7 Departamento de Epidemiología, Unidad de Medicina Familiar N°, 4, Instituto Mexicano del Seguro Social, Guadalajara, Jalisco, México; 8 Laboratorio de Investigación y Desarrollo Farmacéutico, Centro Universitario de Ciencias Exactas e Ingenierías, Universidad de Guadalajara, Guadalajara, Jalisco, México; 9 Unidad de Medicina Familiar No. 2, Instituto Mexicano del Seguro Social, Guadalajara, Jalisco, México; 10 Instituto de Terapéutica Experimental y Clínica, Departamento de Fisiología, Centro Universitario de Ciencias de la Salud, Universidad de Guadalajara, Guadalajara, Jalisco, México; 11 Departamento de Microbiología y Patología, Centro Universitario de Ciencias de la Salud, Universidad de Guadalajara, Guadalajara, México; 12 Departamento de Fisiología, Centro Universitario de Ciencias de la Salud, Universidad de Guadalajara, Guadalajara, México; 13 Departamento de Medicina Interna-Reumatología, Hospital General Regional 110 Instituto Mexicano del Seguro Social, Guadalajara, Jalisco, México; University of Sydney and Westmead Hospital, AUSTRALIA

## Abstract

**Background:**

Only two previous studies in systemic lupus erythematosus (SLE) patients have identified that the blood concentrations of uromodulin are lower in nephritis. However, none of them had evaluated whether a low serum uromodulin adjusted by the glomerular filtration rate (sUromod/eGFR index) contributed to identify patients in risk of lupus nephritis (LN) using multivariable models.

**Aim:**

Therefore, this study aimed two objectives to evaluate the association between low serum uromodulin levels and low sUromod adjusted by eGFR with renal flares in SLE excluding effects of potential confounders in multivariable analyses; and to identify the value of low sUmod and low sUmod/eGFR index as a potential diagnostic marker of LN.

**Patients and methods:**

Design: Cross-sectional study. SLE patients (n = 114) were investigated for lupus flare with renal SLEDAI. Two groups: a) SLE with renal flare (renal-SLEDAI≥4, n = 41) and b) SLE non-renal flare (renal SLEDAI<4, n = 73). SLE patients were evaluated by other indices including a global disease activity index (SLEDAI) and SLICC renal disease activity score. Serum uromodulin levels (ng/mL) were quantified by ELISA. Serum uromodulin was adjusted by eGFR (sUromod/eGFR index). Cutt-offs of low sUromodulin and low sUromod/eGFR index were computed, ROC curves were performed and values of diagnostic tests were obtained. Multivariable logistic regression models were performed to identify if low sUromod/eGFR index is associated to renal flares.

**Results:**

Low serum uromodulin and low sUromod/eGFR index correlated to high scores of renal-SLEDAI, SLICC-renal and proteinuria. SLE patients with a renal flare had lower uromodulin levels compared to SLE patients without renal flare (p = 0.004). After adjusting by potential confounders, the low sUromod/eGFR index (<0.80 ng/mL) increased the risk of a renal flare (OR, 2.91; 95%CI, 1.21 to 6.98; p = 0.02).

**Conclusions:**

We propose the low sUromod/eGFR index as a potential new marker of renal disease activity in SLE.

## Introduction

Lupus nephritis (LN) is a major systemic complication in patients with systemic lupus erythematosus (SLE), with a reported prevalence of up to 70% and an incidence of 38% [1]. The estimated 10-year incidence of end-stage kidney disease (ESKD) in patients with LN is approximately 10% to 20% [1, 2], with a standardized mortality ratio of 5.6 (95% CI 3.7–7.5) [2]. The 10- and 20-year survival rates are also diminished in LN [3]. In many patients with LN, the therapeutic response can be incomplete; only 25% to 30% of patients achieve prolonged remission [4], whereas 24% to 45% of patients may develop new renal flares within two years after remission [5].

New episodes of proteinuria, hematuria, or leukocyturia, or a decrease in creatinine clearance after excluding other causes constitute the clinical characteristics of renal flares [6]. To date, there are a number of biomarkers used for the assessment of renal disease activity, including anti-dsDNA, complement fractions C3 and C4, and more recently, anti-nucleosome antibodies [7, 8]. Our group recently reported in a prospective cohort that the positivity for anti-nucleosome antibodies is associated with an increased risk of development a renal relapse [8]. However, none of these diagnostic markers are highly sensitive in reflecting the presence of renal flares in all SLE patients, and the search for new biomarkers is still ongoing. Uromodulin, previously known as Tamm-Horsfall protein, is a glycoprotein that is synthesized in the ascending limb of Henle’s loop and in the early distal renal tubule. Uromodulin can be detected either in urine or serum [9]. In nonrheumatic patients, several studies have identified low serum uromodulin levels in patients with chronic kidney disease (CKD) [10–13]. However, the role of uromodulin as a biomarker of renal involvement in SLE is unclear. Diminished urine uromodulin levels have been observed in SLE patients with active LN [14]; and other authors have associated abnormal concentrations of urinary Uromodulin with a decline of the renal function in SLE [15]. However, studies performed in non-rheumatic patients have pointed out that serum uromodulin (sUromod) is better than urinary uromodulin to reflect the renal function [14]. Additionally, in those patients with chronic kidney diseases (CKD) of different causes sUromod levels can reflect the tubular function but also other renal parameters, correlating with serum creatinine, blood urea nitrogen and cystatin C [16]. To date, two major studies have been performed to identify if the Uromodulin measured in blood is associated with nephropathy SLE. The first study was performed by Shen et al., reported that the plasma uromodulin levels are significantly diminished in LN or in IgA nephropathy compared to controls [17] and the second work was performed by Scherberich et al., identifying that SLE patients (n = 53) had lower sUromod levels than blood donors and children and these serum levels were even lower in the subgroup with lupus nephritis [16]. However, to date there is insufficient information to identify if the uromodulin measured in serum can be considered as a biomarker for LN. Therefore, this study aimed two objectives the first was to evaluate the association between low serum uromodulin levels and low serum uromodulin adjusted by eGFR with renal flares in SLE excluding effects of potential confounders in multivariable analyses; and to identify the value of low serum uromodulin and low sUmod/eGFR index as a potential diagnostic marker of LN.

## Materials and methods

This cross-sectional study was conducted with 114 patients with SLE who were classified into two groups: a) SLE patients with renal flares (n = 41) and b) SLE patients without renal flares. SLE patients were enrolled at a rheumatology outpatient clinic of a secondary-care center at Regional General Hospital 110 of the Mexican Social Security Institute (IMSS) in Guadalajara, Mexico. For comparison purposes, a control group (CL) comprising 83 patients without preexisting rheumatic diseases who were referred to the same hospital, was included. The controls were similar to the SLE group in age and sex. Prior to their inclusion in this study, all patients signed a voluntary informed consent form. All procedures described in the protocol followed the principles outlined in the Declaration of Helsinki. The study protocol was approved by the Ethics and Research Committee (Registration No. R-2016-1303-6). We included SLE patients if they met the following criteria: 1) met the 1997 American College of Rheumatology criteria for SLE and 2) were ≥18 years of age. The exclusion criteria included the following: 1) patients who were pregnant or breastfeeding; 2) patients with overlap syndrome (coexistence of SLE with other connective tissue diseases, except for Sjögren syndrome); 3) patients with active infections; and 4) patients with other causes of proteinuria, hematuria, or leukocyturia that were not associated with LN, diabetes mellitus-associated nephropathy, urinary tract infections, urinary stones, malignancies, renal insufficiency, or renal transplantation. For the controls, before inclusion in the study, a diagnosis of “clinically healthy” was made by a physician based on a clinical interview, a physical examination and laboratory studies, excluding those with chronic rheumatic diseases, diabetes mellitus, infections, malignancy and other similar criteria that were previously described for SLE patients.

### Clinical assessment

All patients with SLE were assessed by trained researchers. A structured questionnaire and clinical chart review were performed for all the patients, including demographic and SLE characteristics, such as disease duration, comorbidities, history of previous SLE flares, history of LN, and current treatment.

### Assessment of renal function parameters and laboratory parameters of SLEDAI

Serum creatinine, urine creatinine, and total urine protein levels were evaluated for all patients by dry chemistry method (Vitros 3500/4600 analyzer Ortho Diagnostics^TM^). Urinalysis was performed by an experienced researcher to assess the presence of casts, the presence of erythrocyturia (>5 erythrocytes/field), and white blood cell (WBC) counts. The quantification of 24-hour proteinuria (normal range: 0.15 g/day), estimated glomerular filtration rate (eGFR), mL/min/m^2^ body surface, and creatinine clearance (normal range: 100–130 mL/min) were also determined.

### Other laboratory determinations

C3 and C4 were determined in serum by nephelometry (Nephstar Protein Analysis System). Low C3 levels were considered under 90 mg/dL, and low C4 levels under 10 mg/dL. All procedures were performed according to the manufacturer’s recommendations by an experienced researcher. Antibodies against double-stranded DNA (anti-dsDNA) antibodies were identified by indirect immunofluorescence with *Crithidia Luciliae*. An experience observe checked the positivity of anti-dsDNA antibodies.

### Quantification of serum uromodulin levels

Blood samples were taken from all SLE patients and controls on the same day as the clinical evaluation. Serum samples were coded and frozen at -80°C for a maximum of 6 months before the quantification of uromodulin. Serum uromodulin levels were measured by ELISA (Human Uromodulin ELISA, BioVendor—Laboratorí medicina a.s., Karasek, Brno, Czech Republic). The sensitivity for this assay was 0.12 ng/mL, and the intra- and interassay variability were 2.8 and 7.6%, respectively. All measurements were performed by the same researcher, who was blinded to the study groups and to any clinical variables of the patients to minimize measurement bias. For adjusting the serum uromodulin levels by eGFR we computed the index sUromod/eGFR. This index has been previously used elsewhere.

### Assessment of SLE disease activity

The same researchers evaluated SLE disease activity by two methods. The first method was the Systemic Lupus Erythematosus Disease Activity Index (SLEDAI) [18]. The SLEDAI was designed to assess disease activity with 24 weighted clinical and laboratory variables of nine organs/systems. SLEDAI scores can range from 0 to 105. SLE activity was defined as an SLEDAI score ≥ 4 points.

### Assessment of renal flares in SLE patients

Renal flares were identified using the renal SLEDAI (rSLEDAI), which included quantification of proteinuria (>0.5 g/day) or any of the following: the presence of erythrocyturia (> 5 red blood cells per high power field), pyuria (> 5 white blood cells per high power field), or granular or red blood cell casts in urinalysis [18]. Each item was scored with 0 points indicating absence or 4 points indicating presence, and 16 points was the maximum rSLEDAI score. Based on the rSLEDAI score obtained at the time of the study, the SLE patients were classified into two groups. The first group included SLE patients with the presence of renal flares (rSLEDAI scores ≥4), and the second group included SLE patients without renal flares (rSLEDAI scores = 0).

Renal activity was also evaluated by the SLICC (Systemic Lupus International Collaborating Clinics) renal activity score [19]. Briefly, the SLICC renal activity score includes the following renal parameters: proteinuria, the urine red blood cell count, and the urine white blood cell count. The renal activity score was calculated as follows: proteinuria from 0.5 to 1 g/day = 3 points; proteinuria >1 to 3 g/day = 5 points; proteinuria >3 g/day = 11 points; urine red blood cell count >10/hpf = 3 points; and urine white blood cell count >10/hpf = 1 point. The minimum score obtained with this index is equal to 0 points, and the maximum score is equal to 16 points. We arbitrarily classified SLE patients into the following three groups according to the SLICC renal activity score: A) patients with a SLICC renal activity score equal to 0 points; B) patients with SLICC renal activity scores from 1 to 4 points; and C) patients with SLICC renal activity scores ≥ 5 points.

### Evaluation of chronicity

The chronicity of organ damage was assessed with the Systemic Lupus International Collaborating Clinics/American College of Rheumatology (SLICC/ACR) damage index [20].

### Ethics

All procedures described in the protocol followed the principles outlined in the Declaration of Helsinki. The study protocol was approved by the Ethics and Research Committee of the Regional General Hospital 110 of the Mexican Social Security Institute (IMSS) in Guadalajara, Mexico. (Registration No. R-2016-1303-6).

### Statistical analysis

Quantitative variables are described as the means ± standard deviations (SDs), and qualitative variables are described as frequencies (%). To adjust the serum uromodulin levels by eGFR, we computed the sUromod/eGFR index [21]. Spearman tests (Rho) were computed to examine the strength of the association between serum uromodulin concentrations and the sUromod/eGFR index for quantitative variables, such as proteinuria, the eGFR, the creatinine clearance, the rSLEDAI, and the SLICC renal activity score. Chi-square tests (or Fisher’s exact test) were performed for comparisons between proportions. The Mann–Whitney U test was used to compare the medians of quantitative variables between the groups of SLE patients with renal flares and SLE patients without renal flares. The Kruskal-Wallis test was used to compare the serum uromodulin concentrations and sUromod/eGFR index score among the following SLICC renal activity score groups: A) a SLICC renal activity score equal to 0 points; B) SLICC renal activity score from 1 to 4 points; and C) SLICC renal activity score ≥ 5 points. To identify low serum uromodulin levels and low sUromod/eGFR index score, we used the lower quartile (25th percentile) of these measurements that was calculated for the entire group of SLE patients. Receiver Operating Characteristics (ROC) curves were performed to identify the cutoffs with better performance. Sensitivity, specificity, predictive values and likelihood ratios and their 95% confidence intervals were computed using the cutoffs of <83 for low serum uromodulin levels and <0.80 for low sUromod/eGFR index. In the multivariable analysis we performed two logistic regression models. In the first model, we included, as a dependent variable, an rSLEDAI score ≥4 as the definition of renal flare. Age, SLE disease duration, serum creatinine levels, low C3 levels and low C4 levels were included as potential confounders, and a low sUromod/eGFR index score was included as a potential risk factor. The second model was built including a renal SLICC score ≥ 5 as a dependent variable. The same potential confounders described above were included in the second model, and a low sUromod/eGFR index score was included as a potential risk factor. Covariates (potential confounders) included in the regression models were those variables with a p-value ≤0.20 estimated in the univariate analysis or those variables with biological plausibility to the dependent variable. Additionally the variables associated with the risk of renal flares are shown by Method Enter. Odds ratios (ORs) and their 95% confidence intervals (95% CI) were estimated.

Multivariable linear regression analyses were also performed to identify whether potential confounders influence renal flare laboratory parameters. Three models were analyzed: 1) a model with proteinuria (g/day) as the dependent variable; 2) a model with the creatinine clearance as the dependent variable; and 3) a model with the glomerular filtration rate as the dependent variable. In the final model, we included the following renal parameters: proteinuria, creatinine clearance, serum creatinine, and eGFR. We selected the 25th percentile as the cut-off value for low serum uromodulin levels (83 ng/mL). We compared the rSLEDAI and SLICC renal activity scores and proteinuria (g/day) values between SLE patients with serum uromodulin levels below 83 ng/mL (low uromodulin levels) and SLE patients with serum uromodulin levels ≥ 83 ng/mL. We computed the odds ratio (OR) and their 95% confidence intervals (95% CI) for the presence of renal flares in SLE patients with low serum uromodulin levels < 83 ng/mL; we also computed the OR and 95% confidence intervals (CI) for SLE patients with SLICC renal activity score ≥ 1 and serum uromodulin levels < 83 ng/mL. Receiver operating characteristic (ROC) curves were generated to identify risk markers. p-value was set up at 0.05 level. On the analysis was performed using IBM SPSS Statistics software [version 28.0.1.1 (15)].

## Results

Our study included 114 SLE patients, and of these patients, almost 95% were female. The median age for SLE patients was 43.5 years, and at the time of inclusion, the median disease duration of SLE was 10.5 years. Of the total SLE patients included in this study, 73 (63.2%) had previous renal involvement; however, 41 (36.0%) had renal flares at the time of the study. Other organs involved in flares at the time of the study, including clinical and therapeutic characteristics, are shown in Table 1.

**Table 1:** Clinical characteristics of SLE patients at the time of the study.

Variable	n = 114
Age yr, median and ranges	43.5 (18–66)
Female, n (%)	108 (94.7)
**Comorbidities**	
Diabetes, n (%)	13 (11.4)
Hypertension, n (%)	35 (30.7)
Previous renal activity, n (%)	72 (63.2)
**Disease characteristics**	
Disease duration, median and ranges	10.5 (1.0–31.0)
SLE active (SLEDAI ≥4), n (%)	73 (64.0)
SLEDAI score, median and ranges	4.0 (0–26)
rSLEDAI score, median and ranges	0.0 (0.0–16.0)
SLICC renal activity score, median and ranges	0.0 (0.0–12.0)
SLICC renal of 0, n (%)	64 (56.1)
SLICC renal (1–4), n (%)	22 (19.3)
SLICC renal (≥5), n (%)	28 (24.6)
SLICC/ACR, median and ranges	1.0 (0.0–6.0)
**Organs involved**	
Mucocutaneous, n (%)	68 (59.6)
Renal, n (%)	41 (36.0)
Musculoskeletal, n (%)	18 (15.8)
Blood system, n (%)	19 (16.7)
Central nervous system, n (%)	10 (8.8)
**Treatment**	
Glucocorticoids*, n (%)	107 (93.9)
Glucocorticoids Doses (mg/day), score, median and ranges	15.0 (0.0–75.0)
Azathioprine, n (%)	48 (42.1)
Mycophenolate, n (%)	29 (25.4)
Chloroquine, n (%)	28 (24.6)
ACE inhibitors, n (%)	25 (21.9)
Angiotensin II receptor blockers, n (%)	11 (9.6)
**Laboratory parameters**	
Proteinuria (g/day), median and ranges	0.3 (0.04–10.7)
eGFR (mL/min/m^2^), median and ranges	93.8 (14.8–220.9)
Creatinine clearance (mL/min), median and ranges	109.3 (16.53–371.0)
Serum creatinine (mg/dL), median and ranges	0.7 (0.4–3.6)
Positive Anti-dsDNA, n (%)	34 (29.8)
Low C3 levels, n (%)	23 (20.2)
Low C4 levels, n (%)	16 (14)
Serum uromodulin (ng/mL), median and ranges	112.0 (3.0–288.8)
sUromod/eGFR index, median and ranges	1.2 (0.08–5.02)

Qualitative variables are expressed as frequency and percentages. Quantitative variables are expressed as median and ranges. SLE: Systemic lupus erythematosus. SLEDAI: Systemic lupus erythematosus disease activity index. rSLEDAI: renal SLEDAI. SLICC: Systemic Lupus International Collaborating Clinics/American College of Rheumatology damage index. eGFR: estimated glomerular filtration rate. *Glucocorticoids included: prednisone or deflazacort. ACE: Angiotensin-converting enzyme.

We performed a comparison between the SLE patients group and the CL group. No significant differences were observed in age (42 vs. 43 years, respectively, p = 0.3). Furthermore, we observed lower serum uromodulin levels in the SLE patients than in the controls [112.0 (3.0–288.8) ng/mL vs. 146.2 (37.1–598.0) ng/mL, p = 0.003].

Table 2 shows a correlation between serum uromodulin levels and sUromod/eGFR index score with selected variables of the SLE patients. In the uromodulin correlation, we found a negative correlation between serum uromodulin levels and the severity of the SLEDAI disease activity score (Rho = -0.22, p = 0.02). Furthermore, a negative correlation was observed between the rSLEDAI score and serum uromodulin levels (Rho = -0.33, p<0.001). Serum uromodulin levels also showed a negative correlation with the SLICC renal disease activity score (Rho = -0.29, p = 0.002). Regarding the laboratory parameters of renal flares, uromodulin levels had a negative correlation with the severity of proteinuria (Rho = -0.24, p = 0.009), whereas there was a positive correlation between uromodulin levels and creatinine clearance (Rho = 0.23, p = 0.02). A decrease in serum uromodulin levels was correlated with an increase in the SLICC/ACR damage index score (Rho = -0.34, p<0.001). Regarding the sUromod/eGFR index, we found a positive correlation with age (Rho = 0.40, p<0.001) and a negative correlation with SLEDAI (rho = -0.27, p = 0.003), rSLEDAI (Rho = -0.38, p<0.001), renal SLICC (rho = -0.36, p<0.001), SLICC/ACR damage index (Rho = -0.23, p = 0.01) and eGFR values (Rho = -0.21, p = 0.03) [Supporting information].

**Table 2:** Correlations between renal markers and clinical variables.

Variables	Uromodulin ng/mL		sUromod/eGFR index	
	Rho	p	Rho	p
Age, yr	0.17	0.07	0.40	<0.001
BMI (kg/m2)	-0.13	0.18	-0.13	0.19
Disease duration, yr	-0.09	0.33	0.04	0.67
SLEDAI (score)	-0.22	0.02	-0.27	0.003
rSLEDAI (score)	-0.33	<0.001	-0.38	<0.001
Renal-SLICC (score)	-0.29	0.002	-0.36	<0.001
Proteinuria, g/24 h	-0.24	0.009	-0.28	0.003
eGFR (mL/min/m2)	0.19	0.04	-0.21	0.03
Creatinine clearance (mL/min)	0.23	0.02	-0.05	0.58
Serum creatinine, mg	-0.15	0.10	0.16	0.09
SLICC/ACR damage index (score)	-0.34	<0.001	-0.23	0.01

Correlation: Spearmen test (Rho). BMI: body mass index. SLEDAI: Systemic lupus erythematosus disease activity index. rSLEDAI: renal SLEDAI. eGFR: estimated glomerular filtration rate. SLICC/ACR: Systemic Lupus International Collaborating Clinics/American College of Rheumatology damage index

We performed a subanalysis assessing whether serum uromodulin levels were correlated with SLEDAI disease activity index score. We observed that the highest scores of the index were correlated with lower serum uromodulin levels, although after removing the scores given by the renal activity items, the correlation was no longer significant. These findings reflect that serum uromodulin levels are mainly correlated with the renal activity parameter of these indices but are not correlated with other organ involvement parameters.

A comparison between the subgroups of SLE patients with renal flares (n = 41) and patients without renal flares (n = 73) is shown in Table 3. No differences were found in age, sex, disease duration, or the frequency of positive anti-dsDNA antibodies. SLE patients without renal flares had higher uromodulin levels and sUromod/eGFR index score than SLE patients with renal flares (130.9 ng/mL vs. 96.2 ng/mL, p<0.001; and 1.3 vs. 0.8, p<0.001, respectively). No differences were found for other variables.

**Table 3:** Comparison between SLE patients with renal flare and SLE patients without renal flare.

Variable	SLE with renal flare n = 41	SLE without renal flare n = 73	p
Age yr, median and ranges	41.0 (18.0–65.0)	44.0 (18.0–66.0)	0.58
Female, n (%)	38 (92.7)	70 (95.9)	0.46
**Comorbidities**			
Diabetes, n (%)	5 (12.2)	8 (11.0)	0.84
Hypertension, n (%)	15 (36.6)	20 (7.4)	0.31
Previous renal activity, n (%)	41 (100.0)	24 (32.9)	<0.001
**Disease characteristics**			
Disease duration, median and ranges	11.0 (0.5–31.0)	10.0 (0.5–30.0)	0.32
SLICC/ACR	1.0 (0.0–6.0)	0.0 (0.0–6.0)	0.009
**Treatment**			
Glucocorticoids*, n (%)	38 (92.7)	69 (94.5)	0.70
Glucocorticoids Doses (mg/day), median and ranges	15 (0.0–75.0)	10.0 (0.0–75.0)	0.01
Azathioprine, n (%)	15 (36.6)	33 (45.2)	0.37
Mycophenolate, n (%)	13 (31.7)	16 (21.9)	0.25
Chloroquine, n (%)	6 (14.6)	22 (30.1)	0.07
**Laboratory parameters**			
Proteinuria (g/day), median and ranges	1.6 (0.5–10.8)	0.16 (0.04–1.89)	<0.001
eGFR (mL/min/m2), median and ranges	92.3 (14.8–214.2)	93.8 (55.7–220.9)	0.62
Creatinine clearance (mL/min), median and ranges	109.6 (16.5–297.0)	109.0 (28.3–371.0)	0.32
Serum creatinine (mg/dL), median and ranges	0.7 (0.4–3.6)	0.7 (0.4–1.1)	0.28
Positive Anti-dsDNA, n (%)	13 (31.7)	21 (28.8)	0.74
Low C3 levels, n (%)	11 (26.8)	12 (16.4)	0.19
Low C4 levels, n (%)	9 (22.0)	7 (9.6)	0.07
Serum uromodulin (ng/mL), median and ranges	96.2 (3.0–271.3)	130.9 (40.2–288.8)	<0.001
sUromod/eGFR index, median and ranges	0.8 (0.8–2.3)	1.3 (0.4–5.0)	<0.001

Comparison qualitative variables were computed using chi-square test. Comparison of quantitative variables were computed using the U-Mann Whitney test. SLEDAI: Systemic lupus erythematosus disease activity index. SLICC/ACR: Systemic Lupus International Collaborating Clinics/American College of Rheumatology

**Fig 1:**
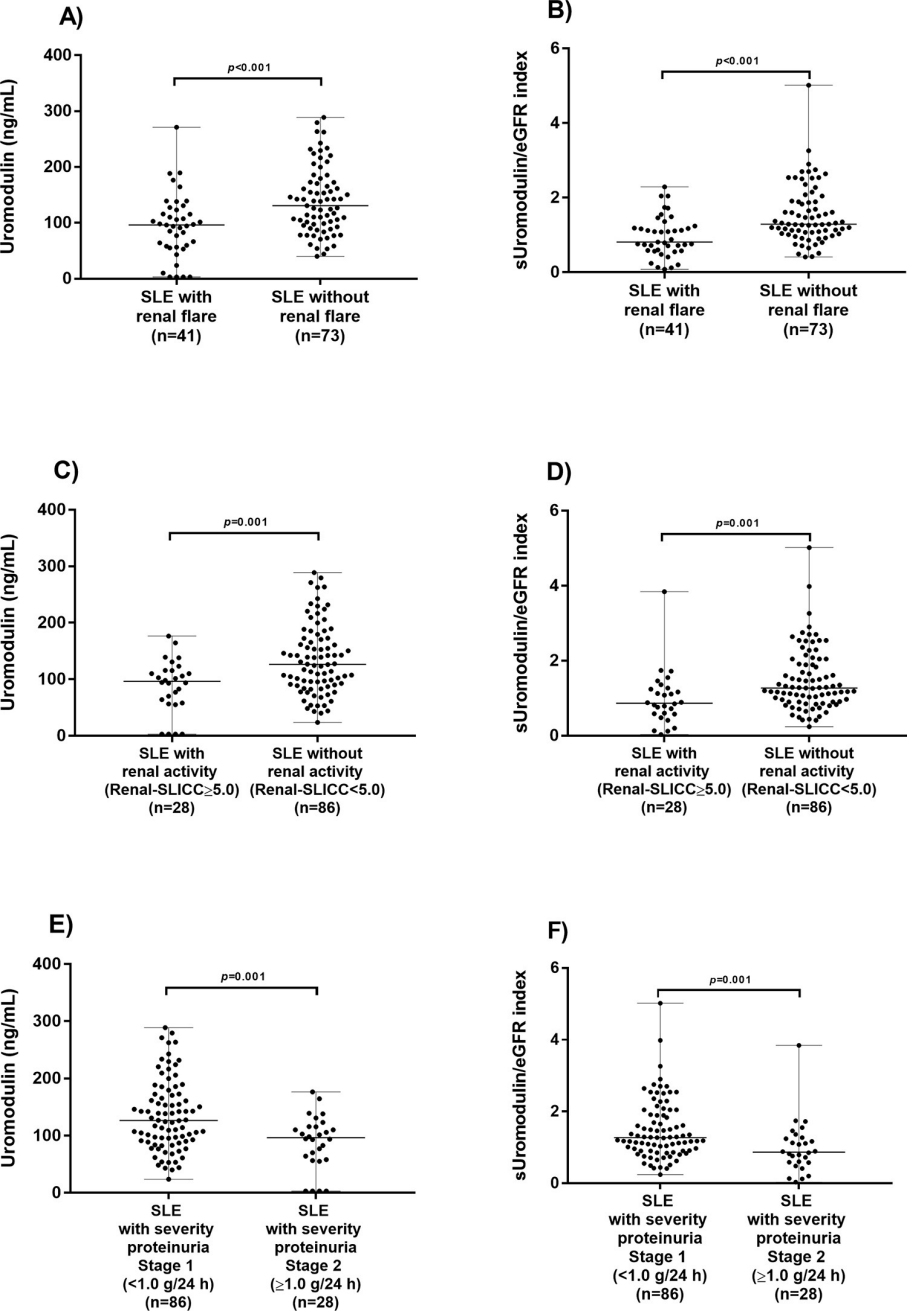
Comparison of uromodulin levels and sUromod/eGFR index score between SLE patients with renal flare and SLE patients without renal flare. (A) Comparison of serum uromodulin levels between SLE patients with renal flare and SLE patients without renal flare by renal SLEDAI score (cut-off ≥ 4.0). (B) Comparison of sUromod/eGFR index score between SLE patients with renal flare and SLE patients without renal flare by renal SLEDAI score (cut-off < 4.0). (C) Comparison of serum uromodulin levels between SLE patients with renal activity and SLE patients without renal activity by renal SLICC score (cut-off ≥ 5.0). (D) Comparison of sUromod/eGFR index score between SLE patients with renal activity and SLE patients without renal activity by renal SLICC score (cut-off < 5.0). (E) Comparison of serum uromodulin levels between SLE patients with a proteinuria severity of stage 1 (<1.0 g/24 h) and SLE patients with a proteinuria severity of stage 2 (≥1.0 g/24 h). (F) Comparison of sUromod/eGFR index score between SLE patients with a proteinuria severity of stage 1 (<1.0 g/24 h) and SLE patients with a proteinuria severity of stage 2 (≥1.0 g/24 h). The comparison was performed using the Mann–Whitney U test.

We observed lower serum uromodulin levels in SLE patients with renal activity, with respect to those without renal activity, using a cut-off renal SLICC score of ≥ 5.0 (p<0.001), in the same way that we observed a lower sUromod/eGFR index score in SLE patients with renal activity (p<0.001). In addition, we observed higher serum uromodulin levels in SLE patients with a proteinuria severity of stage 1 (<1.0 g/24 h) compared with a proteinuria severity of stage 2 (≥1.0 g/24 h) (p = 0.001), and higher sUromod/eGFR index scores (p = 0.001) (Fig 1).

### Renal activity by SLICC renal score was associated with low uromodulin levels

We compared serum uromodulin levels defined by the SLICC renal activity score among the following three subgroups: A) SLE patients with a SLICC renal activity score equal to 0 points (n = 63); B) SLE patients with SLICC renal activity score from 1 to 4 points (n = 23); and C) SLE patients with SLICC renal activity score ≥ 5 points (n = 28).

**Fig 2:**
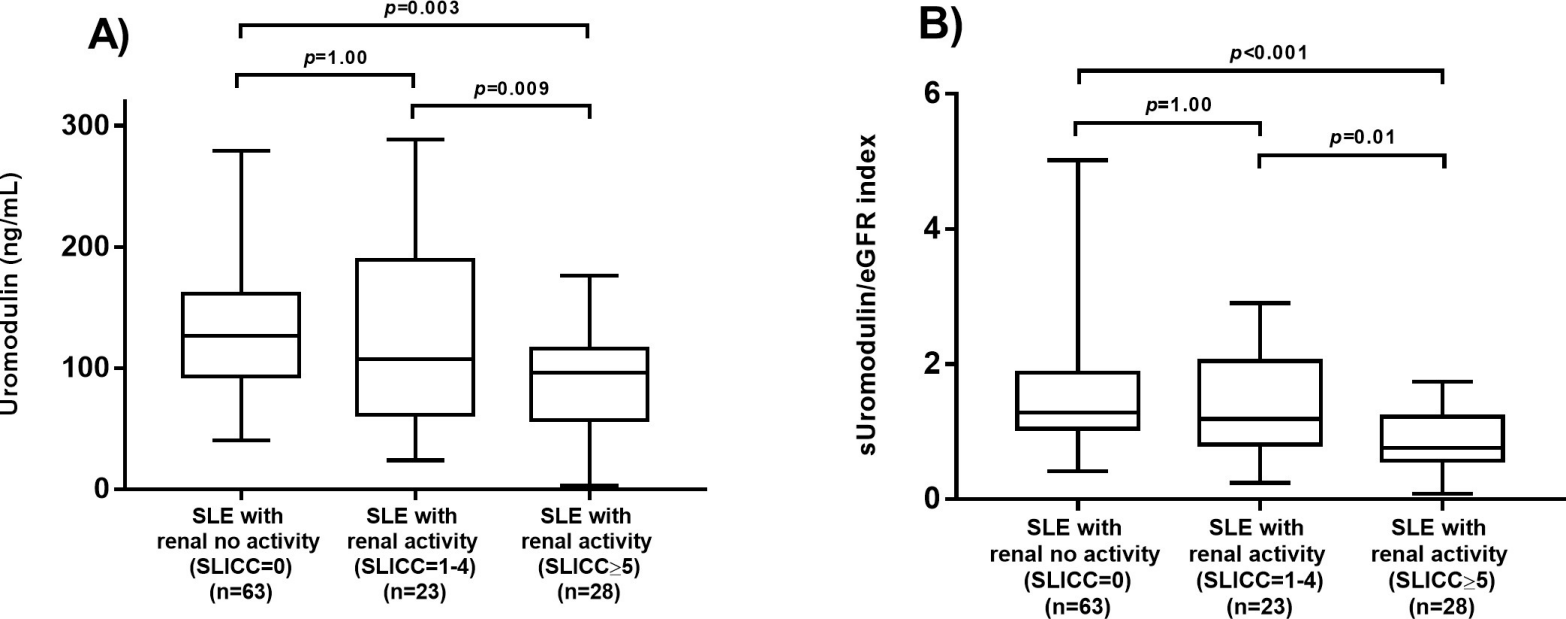
Comparison of serum uromodulin levels and sUromod/eGFR index between SLE patients with a SLICC renal activity score. (A) Comparison of serum uromodulin levels between renal activities by SLICC score. (B) Comparison of sUromod/eGFR index score between renal activities by SLICC score. The comparisons were performed by the Kruskal–Wallis test.

Patients with SLICC renal activity score ≥ 5 points had lower titers of uromodulin (p = 0.003) and lower sUromod/eGFR scores (p<0.001) than patients with a SLICC renal activity score equal to 0 points. Additionally, SLE patients with a SLICC renal activity score ≥ 5 points had lower sUromod/eGFR scores (p = 0.01) than SLE patients with a SLICC renal activity score from 1 to 4 points. However, no differences were observed between SLE patients with a SLICC renal activity score from 1 to 4 points versus those with a SLICC renal activity score equal to 0 points (p = 1.0) (Fig 2).

**Fig 3:**
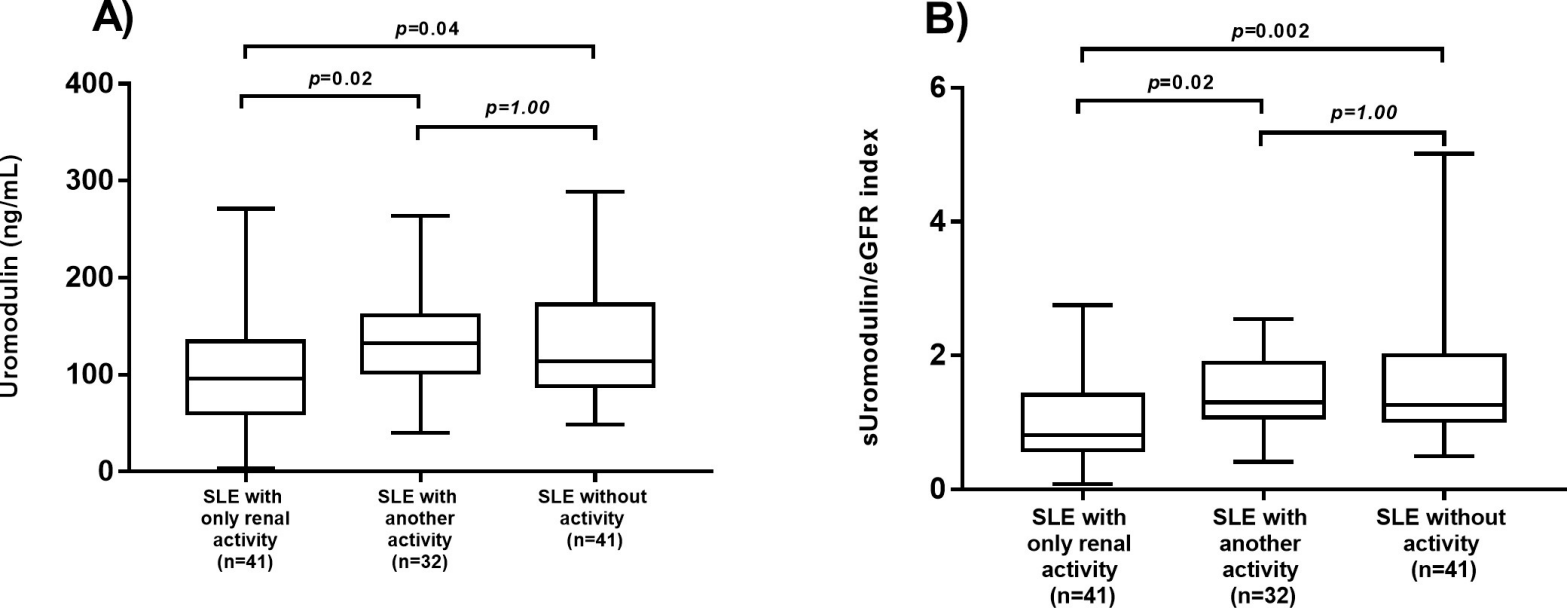
Comparison of serum uromodulin levels and sUromod/eGFR index score among SLE patients with renal activity, other activity and inactivity. (A) Comparison of serum uromodulin levels among patients with renal activity, other activity and inactivity. (B) Comparison of sUromod/eGFR index score among patients with renal activity, other activity and inactivity. The comparisons were performed by the Kruskal–Wallis test.

Statistical differences in serum uromodulin levels and sUromod/eGFR index among patients with only renal activity (n = 41), patients with another type of activity (n = 32) and inactive patients (n = 41) were found. SLE patients with only renal activity had lower serum uromodulin levels (p = 0.02) and lower sUromod/eGFR index score (p = 0.02) than SLE patients with other involved organs (different from renal flares) (Fig 3).

### Risk of renal flares associated with low uromodulin levels

The group of SLE patients with serum uromodulin levels <83 ng/mL (n = 29) had higher rSLEDAI scores (6.0 vs. 4.0; p = 0.01), higher SLICC renal activity scores (3.0 vs. 0.0; p = 0.01), and higher proteinuria values (0.6 g/24 h vs. 0.3 g/24 h; p = 0.05) than the group of SLE patients with serum uromodulin levels ≥ 83 ng/mL (n = 85).

### Utility values of low serum uromodulin levels (cut-off<83.0 ng/mL) and low sUromod/eGFR index (cut-off<0.80) to identify SLE patients with renal flares

S2 Table shows the utility values of serum uromodulin levels and sUromod/eGFR index in Lupus nephritis and SLE patients (renal SLEDAI ≥ 4). Using the previously referred cut-off points the sensitivity of low serum uromodulin levels for detecting renal flares, was 39.0% with a specificity of 82.2%; whereas the sensitivity of low sUromod/eGFR index was 48.8% with a specificity of 87.7%. Other utility values are shown in that table.

### Risk of renal flares associated with a low index score for uromodulin levels adjusted by the eGFR

Table 4 shows the risk of renal flares, after adjusting for age, SLE disease duration, low C4 levels, low C3 levels, and anti-dsDNA antibody positivity, the logistic regression models showed that SLE patients with a low index score for serum uromodulin levels adjusted by the eGFR (sUromod/eGFR index score < 0.80) had an increased risk of renal flares identified by the renal SLEDAI (OR 2.91 95% CI 1.21 to 6.98; p = 0.02). Similarly, after adjusting for age, disease duration, C3 levels, C4 levels and anti-dsDNA antibody positivity, SLE patients with a low sUromod/eGFR index score had an increased risk of having a SLICC renal activity score ≥ 5 (OR 4.27, 95% CI 1.60 to 11.38; p = 0.004).

**Table 4:** Risk of renal flares associated with uromodulin adjusted by eGFR.

Renal SLEDAI							SLICC RENAL						
Variable	Method Enter			Method Forward			Variable	Method Enter			Method Forward		
	OR	95% CI	p	OR	95% CI	p		OR	95% CI	p	OR	95% CI	p
Age	0.99	0.96 to 1.01	0.43	Not in the model	-	-	Age	0.96	0.92 to 0.99	0.01	Not in the model	-	-
SLE disease duration	1.02	0.97 to 1.07	0.37	Not in the model	-	-	SLE disease duration	0.98	0.92 to 1.04	0.50	Not in the model	-	-
Low C3 levels	0.37	0.14 to 0.96	0.04	Not in the model	-	-	Low C3 levels	0.32	0.12 to 0.84	0.02	Not in the model	-	-
Low C4 levels	0.30	0.10 to 0.91	0.03	Not in the model	-	-	Low C4 levels	0.19	0.06 to 0.56	0.003	Not in the model	-	-
Positive Anti-dsDNA	1.46	0.65 to 3.32	0.36	Not in the model	-	-	Positive Anti-dsDNA	1.77	0.72 to 4.34	0.21	Not in the model	-	-
sUromod/eGFR index < 0.80	5.99	2.38 to 15.04	0.001	2.91	1.21 to 6.98	0.02	sUromod/eGFR index < 0.80	7.49	2.88 to 19.41	0.001	4.27	1.60 to 11.38	0.004

Multivariate analysis was performed by logistic regression. Models adjusted by age, SLE disease duration, Low C3 levels, Low C4 levels, positive Anti-dsDNA and serum uromodulin adjusted by eGFR< 0.80

### Uromodulin and other factors associated with renal function parameters

The multiple linear regression analyses tested whether parameters of renal function (proteinuria, creatinine clearance, and glomerular filtration rate) were associated with uromodulin by adjusting for potential confounders. The multiple regression models are shown in Table 5. For the model assessing variables related to proteinuria (g/day), we identified an association with lower levels of uromodulin (p = 0.009), younger age (p = 0.012), and a history of previous renal involvement (p = 0.009). For the model assessing variables related to creatinine clearance, an association was observed with higher serum uromodulin levels (p<0.001) and older age (p<0.001). For the model assessing variables related to the eGFR, an association was found with younger age (p<0.001).

**Table 5:** Multivariable linear regression models associated with uromodulin.

Independent Variables	Model A				Model B				Model C			
	Proteinuria (g/day)				Creatinine clearance (mL/min)				eGFR (mL/min/m^2^)			
	Unadjusted		Adjusted		Unadjusted		Adjusted		Unadjusted		Adjusted	
	β coefficient (95%CI)	p	β coefficient (95%CI)	p	β coefficient (95%CI)	p	β coefficient (95%CI)	p	β coefficient (95%CI)	p	β coefficient (95%CI)	p
Age	-0.03 (-0.06 to 0.001)	0.06	-0.03 (-0.05 to -0.01)	0.012	-2.29 (-3.05 to -1.52)	<0.001	-2.56 (-3.22 to -1.89)	<0.001	-1.00 (-1.49 to -0.52)	<0.001	-1.27 (-1.68 to -0.87)	<0.001
Glucocorticoids Doses	0.06 (0.04 to 0.08)	<0.001	0.06 (0.04 to 0.08)	<0.001	-0.52 (-1.07 to 0.03)	0.06	-0.55 (-1.09 to -0.17)	0.04	-0.25 (-0.59 to 0.09)	0.16	-0.42 (-0.75 to -0.10)	0.01
Uromodulin	-0.006 (-0.01 to -0.001)	0.03	-0.006 (-0.01 to -0.001)	0.009	0.22 (0.07 to 0.37)	0.004	0.24 (0.10 to 0.37)	<0.001	0.12 (0.02 to 0.21)	0.02	Excluded in the adjusted model	
Previous renal activity	0.72 (0.06 to 1.38)	0.03	0.81 (0.19 to 1.42)	0.009	-7.00 (-25.00 to 10.99)	0.44	Excluded in the adjusted model		-1.03 (-12.38 to 10.31)	0.86	Excluded in the adjusted model	
SLICC/ACR	-0.12 (-0.35 to 0.10)	0.29	Excluded in the adjusted model		-3.86 (-10.03 to 2.31)	0.22	Excluded in the adjusted model		-2.68 (-6.56 to 1.21)	0.18	-5.11 (-8.55 to -1.68)	0.004
SLEDAI score	0.06 (-0.005 to 0.12)	0.07	Excluded in the adjusted model		0.09 (-1.65 to 1.83)	0.92	Excluded in the adjusted model		0.08 (-1.02 to 1.18)	0.89	Excluded in the adjusted model	
Azathioprine	0.05 (-0.67 to 0.77)	0.89	Excluded in the adjusted model		1.56 (-18.11 to 21.23)	0.86	Excluded in the adjusted model		0.36 (-12.03 to 12.76)	0.95	Excluded in the adjusted model	
Mycophenolate	0.33 (-0.50 to 1.17)	0.43	Excluded in the adjusted model		21.54 (-1.32 to 44.39)	0.06	Excluded in the adjusted model		7.69 (-6.70 to 22.10)	0.23	Excluded in the adjusted model	
Disease duration	0.009 (-0.04 to 0.06)	0.69	Excluded in the adjusted model		-0.51 (1.76 to 0.74)	0.42	Excluded in the adjusted model		-0.28 (-1.07 to 0.51)	0.49	Excluded in the adjusted model	

The models were adjusted age, glucocorticoids use, previous renal activity, SLICC/ACR, SLEDAI score, Azathioprine use, Mycophenolate use and disease duration. SLEDAI score, azathioprine, mycophenolate and disease duration were not significant in the adjusted models (Therefore were excluded from the final model). Previous renal activity was significant in the model for proteinuria; and SLICC/ACR was significant in the model for eGFR). Excluded AM: Excluded in the adjusted model.

## Discussion

This study identified an association between low serum uromodulin levels and renal flare parameters in LN. Additionally, after the adjustment of potential confounders, a low sUromod/eGFR index score was significantly associated with a higher risk of LN in the multivariable approach.

However, studies performed in other populations have shown that serum uromodulin better reflects the renal function parameters than urinary uromodulin [14]. In SLE patients, most of the studies assessing the association between renal flares and uromodulin levels have been performed testing urinary uromodulin [15, 21]. To the best of our knowledge, only a few studies have evaluated the correlation between uromodulin measured in blood and clinical parameters of renal involvement in SLE patients [16, 17]. In one study, Shen et al., assessed 36 SLE patients, 58 IgA nephropathy patients and 30 healthy controls; after pooling all the included individuals, these authors identified a correlation of plasma uromodulin with the eGFR (r = 0.255; P = 0.013), serum creatinine (r = ‐0.307; P = 0.003) and other parameters of renal function. In a second study, Scherberich et al. [16] investigated 132 patients with CKD (of them, only 53 were SLE patients), 190 adult blood donors and 443 children and adolescents; these authors pooled all the groups with different diseases that might potentially affect renal function, and identified a correlation of serum uromodulin with the eGFR adjusted by cystatin C (r = 0.842; p<0.001), serum creatinine (r = ‐0.802; p<0.001) and other parameters of renal function. Similar to these studies, we found a correlation between blood levels of uromodulin and the eGFR, creatinine, or creatinine clearance. However, none of these previous studies reported a correlation between serum or plasma uromodulin levels and proteinuria. Proteinuria is a well-known major marker of renal involvement in SLE and is included in the main indices for identifying renal flares, such as the renal SLEDAI and renal SLICC. Proteinuria is also a marker of renal function prognosis in SLE patients, identifies persistent renal activity and is associated with cardiovascular morbidity [22, 23]. Therefore, the investigation of markers associated with renal proteinuria is required in patients with LN. In our patients, we identified that lower levels of serum uromodulin were correlated with the intensity of proteinuria, and this correlation was also observed after adjusting serum uromodulin by the eGFR using the sUromod/eGFR index. This adjustment of serum uromodulin by the eGFR has been utilized by others in other populations [24].

We identified an association between renal flares in patients with low uromodulin levels and a low sUromod/eGFR index score in the univariable analysis (Table 2). In the multivariable logistic regression analysis, the risk of lupus flare identified by the renal SLEDAI was increased in patients with a low sUromod/eGFR index score (OR = 3.08, 95% CI 1.18 to 8.02, p = 0.02) and a higher risk of a SLICC renal activity score ≥ 5 (OR 4.27, 95% CI 1.60 to 11.38; p = 0.004).

These data are supported by Scherberich et al., who identified, in a subanalysis of CKD patients, that patients with SLE and renal involvement had lower serum uromodulin levels than SLE patients without renal involvement and controls [16].

We investigated the association between serum uromodulin levels and the main renal disease activity indices in SLE patients, such as the renal SLEDAI or SLICC renal disease activity scores. Bombardier et al. proposed the utilization of the rSLEDAI to help clinicians identify renal activity (or renal flares) in the last ten days [18]. A more recent renal disease activity index is the SLICC renal activity score; this instrument has been validated to assess renal activity [19]. Only one study by Bedair et al. evaluated the correlation between the SLICC renal activity score and urinary uromodulin, and the findings showed that a low urinary uromodulin level was correlated with a high SLICC renal activity score [21]. Therefore, our study is the first to identify the association between low serum uromodulin levels or a low sUromod/eGFR index score and renal flares assessed by the rSLEDAI or a SLICC renal activity score ≥ 5 in SLE patients.

In SLE patients, renal flares have been correlated with a worse renal outcome [25]. Renal involvement is associated with an increased standardized mortality rate of 7.9%, and this mortality rate increased to 26% in SLE patients who developed ESKD [26]. Continuous fluctuations between remission and renal flares lead to the development of glomerulosclerosis, tubular atrophy, and tissue fibrosis [27, 28]. In the clinical scenario, one of the main parameters for the suspicion of renal involvement is proteinuria, which is a helpful biomarker that is used by clinicians to adjust treatment strategies in SLE patients. Proteinuria is also a key finding that is included as a laboratory variable in the indices for evaluating the severity of renal disease, such as renal SLEDAI and SLICC renal disease activity scores [18, 19]. Therefore, new biomarkers that correlate with proteinuria or other clinical and laboratory features related to renal involvement should be investigated.

### Comparison of studies assessing uromodulin in blood with our results

Uromodulin is a glycoprotein that is synthesized mainly in the ascending limb of Henle’s loop in the kidney, and it can be detected either in urine or serum. The role of uromodulin as a biomarker of kidney function has been supported by experimental and clinical studies. Bachmann et al., in a uromodulin knockout mice model, identified that the absence of the uromodulin gene was associated with a reduction in creatinine clearance and other variables reflecting renal impairment [29]. Alfaham et al. observed the presence of low serum uromodulin levels in children with renal disease attributed to different causes compared to children without renal disease in the reference group [30]. Scherberich et al. identified lower serum uromodulin levels in patients with CKD secondary to different diseases, including a group of SLE patients, than in controls without renal disease [16]. In the same manner, serum uromodulin has been linked to tubular atrophy in glomerulopathies that are not associated with SLE. Smirnov et al. observed earlier changes in serum uromodulin levels in patients with a recent onset of tubular atrophy [31]. Serum uromodulin is an early biomarker for tubular atrophy and interstitial fibrosis in patients with glomerulopathies [31].

No previous studies have been published that assess the risk of low serum uromodulin levels adjusted by the eGFR for renal relapses. In this study, the unadjusted analysis identified that the presence of serum uromodulin levels < 83 ng/mL is associated with a 3-fold risk of renal flare. In the multivariable analysis after adjusting serum uromodulin by the eGFR, the risk of renal flare by the rSLEDAI was 2.94, independent of other variables, whereas the risk increased to 4.27 when a patient with flare had a SLICC renal disease activity score of ≥5.

### Study strengths

Our study has several strengths that should be identified. First, the researchers who evaluated the serum uromodulin levels were blinded to any clinical or laboratory characteristics of the SLE patients. This strategy could minimize the probability of expectancy bias that could deviate the true magnitude of the results. The second strength that should be pointed out is that the present study included the evaluation of serum uromodulin adjusted by the eGFR to identify its correlation with a wide spectrum of parameters of renal involvement and renal dysfunction, including proteinuria, creatinine clearance, and serum creatinine, which are commonly used in the clinic for detecting renal flares in SLE patients. Third, we assessed the association of serum uromodulin levels and serum uromodulin levels adjusted by the eGFR with two renal disease activity indices, the rSLEDAI and the SLICC renal activity score, identifying that low serum uromodulin levels and a low sUromod/eGFR index score are associated with renal involvement. Fourth, this study is the first to use a multivariable logistic regression analysis to adjust for potential confounders, demonstrating that a low sUromod/eGFR index score is associated with an increase in ORs, meaning that this index can be used as a potential marker of the risk of renal flare. Based on these results, we propose that the sUromod/eGFR index can be used as a potential biomarker in the evaluation of renal flares in SLE patients.

### Biologic plausibility

To date it is unknown the mechanisms of uromodulin to protect the renal function. Experimental studies using uromodulin knockout mice reveal a protective role for this protein in acute kidney injury, down-regulating interstitial inflammation [29]. Some authors have described that uromodulin excretion adjusted for kidney function increased reactively to injury, and reflects an increase of uromodulin in the renal parenchyma. Uromodulin has potential anti-inflammatory or pro-inflammatory effects depending of the cells types within and urinary tract [31].

### Study limitations

Our study has several limitations that should be considered. The cross-sectional design of our study was limited in its ability to establish a causal association between uromodulin levels and the development of renal flares in SLE patients. Nonetheless, this finding supports the potential utility of serum uromodulin levels for identifying renal flares. Follow-up studies should be performed to identify if changes in the levels of this molecule precede a renal flare. Another limitation of our study is that we included SLE patients with a long disease duration; therefore, we propose the potential use of this biomarker as a new parameter of renal flares, but we have no information regarding serum levels of uromodulin in recent-onset SLE patients. Further studies are required to identify the value of serum uromodulin levels in early SLE. Moreover, the results of this study raise new hypotheses for longitudinal studies to determine whether the decrease in serum uromodulin levels or renal flares appears first in the clinical and pathological course of the disease. Finally, another limitation in the present study was the lack of renal biopsy in these SLE patients at the time of the study. Although the diagnosis of renal flare relies on laboratory parameters and the validated clinical indices, such as the SLICC renal activity score and the renal SLEDAI [32], has been identified as a valid assessment, we were unable to evaluate a correlation between serum uromodulin levels or sUromod/eGFR index score with the histopathological findings. Further studies should address this limitation.

## Conclusion

In conclusion, low serum uromodulin levels and low sUromod/eGFR index scores were associated with the main parameters of renal flares, such as proteinuria, creatinine clearance, and serum creatinine in SLE patients as well as the diagnosis of flares by the renal SLEDAI. These associations remained after excluding potential confounders in the multivariable analyses. Therefore, low serum uromodulin levels adjusted by the eGFR can be considered a marker associated with renal flares. However, longitudinal studies that include patients with new-onset SLE and treatment-naive patients are needed.

## Supporting information

S1 Fig Correlations between serum uromodulin levels and clinical variables. **S1 Fig** shows the correlations of serum uromodulin levels with clinical variables in SLE patients: (A) correlation between serum uromodulin levels and age; (B) correlation between serum uromodulin levels and disease activity by SLEDAI score; (C) correlation between serum uromodulin levels and disease activity with renal domain by rSLEDAI score; (D) correlation between serum uromodulin levels and renal SLICC scores; (E) correlation between serum uromodulin levels and proteinuria; (F) correlation between serum uromodulin levels and the glomerular filtration rate (eGFR); (G) correlation between serum uromodulin levels and creatinine clearance; and (H) correlation between serum uromodulin levels and SLICC/ACR damage index scores. Correlations were examined by Spearman’s test (Rho).
(TIF)

S2 Fig Correlations between sUromod/eGFR index scores and clinical variables. **S2 Fig** shows the correlations of serum sUromod/eGFR index scores with clinical variables in SLE patients: (A) correlation between sUromod/eGFR index scores and age; (B) correlation between sUromod/eGFR index scores and disease activity by SLEDAI score; (C) correlation between sUromod/eGFR index scores and disease activity with renal domain by rSLEDAI score; (D) correlation between sUromod/eGFR index scores and renal SLICC scores; (E) correlation between serum uromodulin levels and proteinuria; (F) correlation between sUromod/eGFR index scores and the glomerular filtration rate (eGFR); (G) correlation between sUromod/eGFR index scores and serum creatinine levels; and (H) correlation between sUromod/eGFR index scores and SLICC/ACR damage index scores. Correlations were examined by Spearman’s test (Rho).
(TIF)

S3 Fig Shows the Receiver Operating Characteristics (ROC) curves were performed to identify the cutoffs with better performance. Sensitivity, specificity, predictive values and likelihood ratios and their 95% confidence intervals were computed using the cutoffs of <83 for low serum uromodulin levels and <0.80 for low sUromod/eGFR index.
(TIF)

S1 Table ROC curve analysis of different potential biomarkers of Lupus nephritis defined by renal SLEDAI ≥ 4 or renal SLICC ≥ 5. (DOCX)

S2 Table Utility values of serum uromodulin levels and sUromod/eGFR index in Lupus nephritis and SLE patients (renal SLEDAI ≥ 4). (DOCX)

S1 Dataset (CSV)
