# Supplementary figures and images for: Low serum uromodulin levels and their association with lupus flares
Source: PLoS One. 2022 Oct 27;17(10):e0276481. doi: 10.1371/journal.pone.0276481 (PMC9612514; doi:10.1371/journal.pone.0276481)

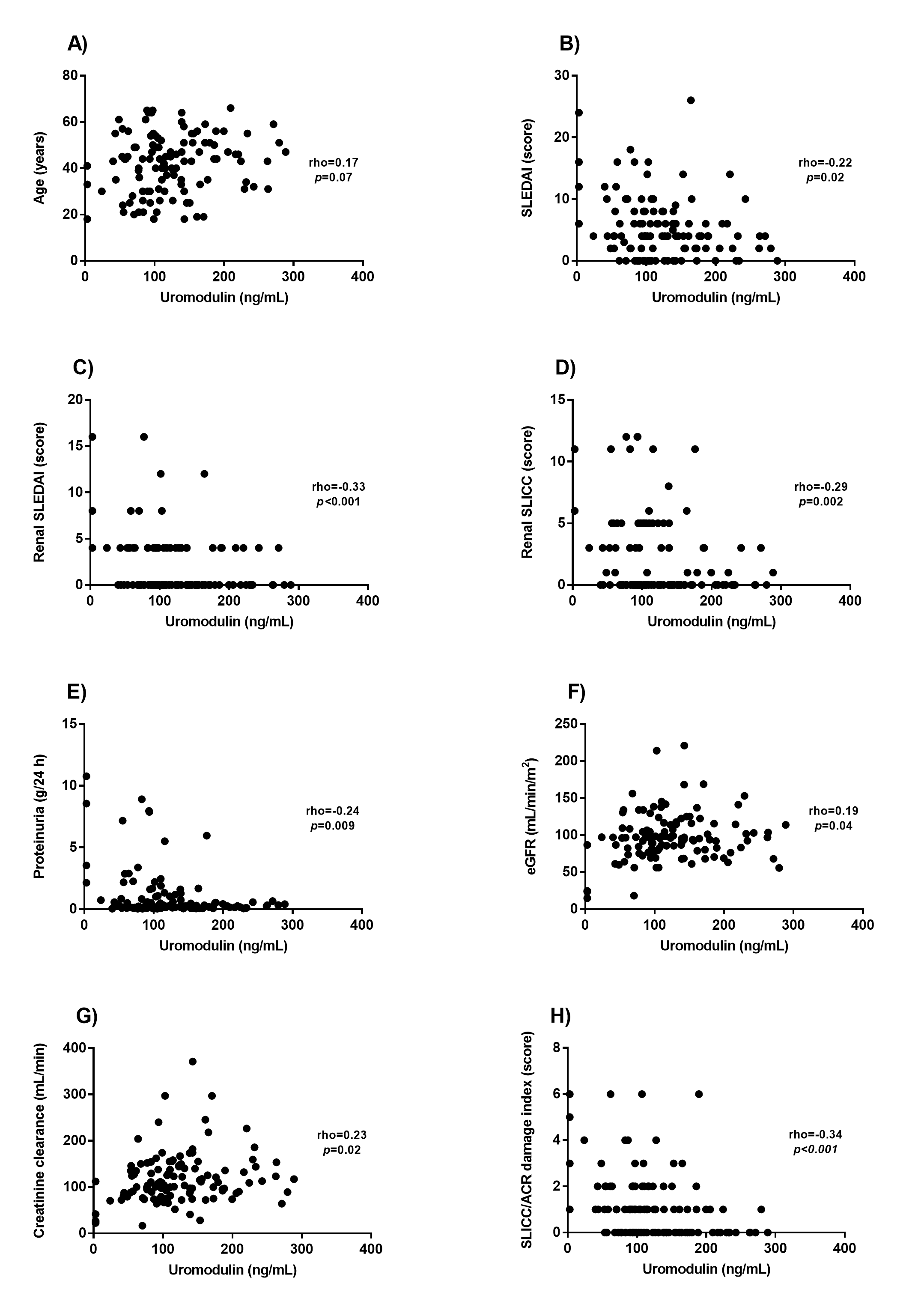

Supplement: S1 Fig — shows the correlations of serum uromodulin levels with clinical variables in SLE patients: (A) correlation between serum uromodulin levels and age; (B) correlation between serum uromodulin levels and disease activity by SLEDAI score; (C) correlation between serum uromodulin levels and disease activity with renal domain by rSLEDAI score; (D) correlation between serum uromodulin levels and renal SLICC scores; (E) correlation between serum uromodulin levels and proteinuria; (F) correlation between serum uromodulin levels and the glomerular filtration rate (eGFR); (G) correlation between serum uromodulin levels and creatinine clearance; and (H) correlation between serum uromodulin levels and SLICC/ACR damage index scores. Correlations were examined by Spearman’s test (Rho). (TIF) [file pone.0276481.s001.tif]

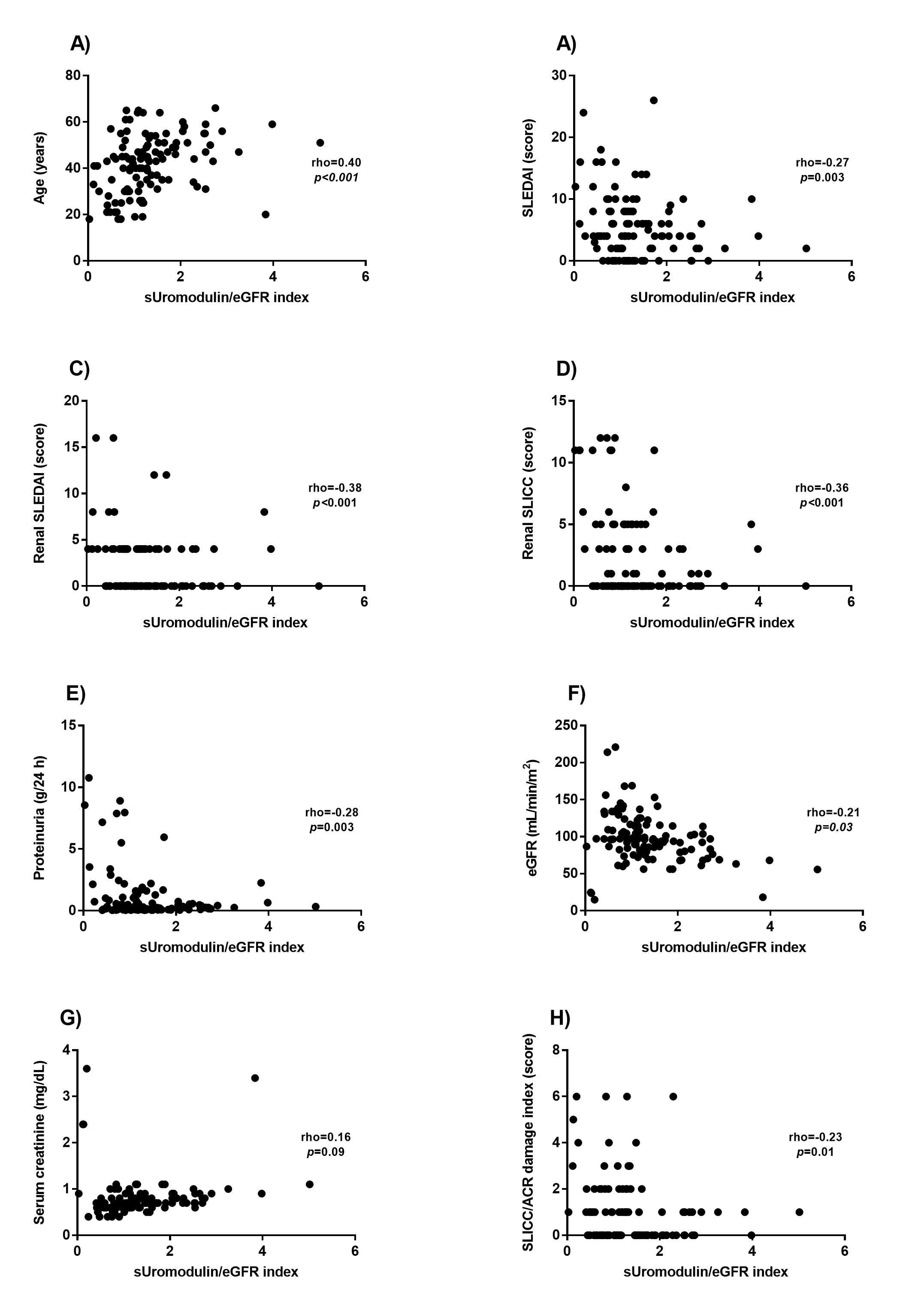

Supplement: S2 Fig — shows the correlations of serum sUromod/eGFR index scores with clinical variables in SLE patients: (A) correlation between sUromod/eGFR index scores and age; (B) correlation between sUromod/eGFR index scores and disease activity by SLEDAI score; (C) correlation between sUromod/eGFR index scores and disease activity with renal domain by rSLEDAI score; (D) correlation between sUromod/eGFR index scores and renal SLICC scores; (E) correlation between serum uromodulin levels and proteinuria; (F) correlation between sUromod/eGFR index scores and the glomerular filtration rate (eGFR); (G) correlation between sUromod/eGFR index scores and serum creatinine levels; and (H) correlation between sUromod/eGFR index scores and SLICC/ACR damage index scores. Correlations were examined by Spearman’s test (Rho). (TIF) [file pone.0276481.s002.tif]

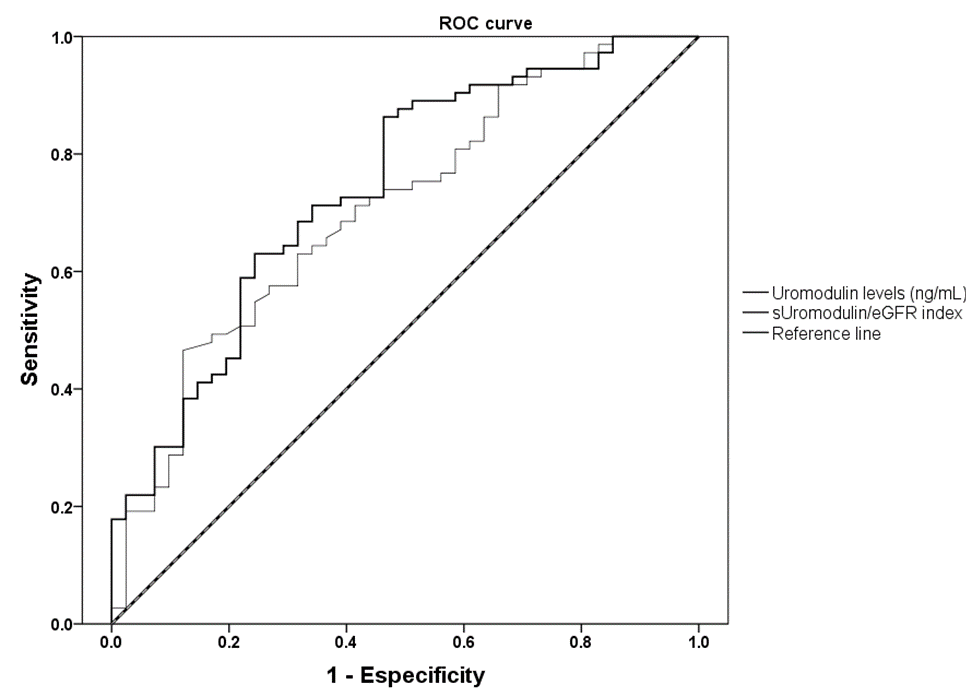

Supplement: S3 Fig — Sensitivity, specificity, predictive values and likelihood ratios and their 95% confidence intervals were computed using the cutoffs of <83 for low serum uromodulin levels and <0.80 for low sUromod/eGFR index. (TIF) [file pone.0276481.s003.tif]
