# Supplementary material for: Low serum uromodulin levels and their association with lupus flares
Source: PLoS One. 2022 Oct 27;17(10):e0276481. doi: 10.1371/journal.pone.0276481 (PMC9612514; doi:10.1371/journal.pone.0276481)
Supplement: S1 Table — (DOCX) [file pone.0276481.s004.docx]

**Supplementary table 1. ROC curve analysis of different potential biomarkers of Lupus nephritis defined by renal SLEDAI ≥ 4 or renal SLICC ≥ 5.**

| Renal SLEDAI >4 | Cutoff | AUC (%) | Sensitivity (%) | Specificity (%) |
| --- | --- | --- | --- | --- |
| Low serum uromodulin | 79.80 | 66.2 | 62.5 | 74.2 |
| Proteinuria | 1.785 | 68.4 | 65.7 | 75.0 |
| Serum creatinine | 0.850 | 60.4 | 54.2 | 75.0 |
| eGFR | 90.70 | 61.1 | 51.0 | 50.0 |
| low sUromod/eGFR | 0.65 | 61.1 | 62.0 | 82.0 |
| renal SLICC >5 | 0.16 | 70.5 | 67.8 | 58.2 |
| AUC: Area Under the Curve | | | | |
