# Supplementary material for: Low serum uromodulin levels and their association with lupus flares
Source: PLoS One. 2022 Oct 27;17(10):e0276481. doi: 10.1371/journal.pone.0276481 (PMC9612514; doi:10.1371/journal.pone.0276481)
Supplement: S2 Table — (DOCX) [file pone.0276481.s005.docx]

**Supplementary table 2. Utility values of serum uromodulin levels and sUromod/eGFR index in Lupus nephritis and SLE patients (renal SLEDAI ≥ 4)**

| Utility values | Low serum  uromodulin | low sUromod/eGFR index |
| --- | --- | --- |
| Cutoff | <83.0 ng/mL | <0.80 |
| AUC % (95% CI) | 0.61 (0.51-0.70) | 0.68 (0.59-0.77) |
| Sensitivity % (95% CI) | 39.0 (24.2-55.5) | 48.8 (32.8-64.9) |
| Specificity % (95% CI) | 82.2 (71.5-90.2) | 87.7 (77.9-94.2) |
| Positive predictive value % (95% CI) | 55.2 (39.8-69.7) | 68.9 (52.8-81.6) |
| Negative predictive value % (95% CI) | 70.6 (64.7-75.8) | 75.3 (69.0-80.6) |
| LR+ (95% CI) | 2.2 (1.2-4.1) | 3.9 (1.9-7.9) |
| LR- (95% CI) | 0.7 (0.6-0.9) | 0.6 (0.4-0.8) |
| AUC: Area Under the Curve; LR+: positive likelihood ratio; LR-: negative likelihood ratio. | | |
